# Effect of Substance P in *Staphylococcus aureus* and *Staphylococcus epidermidis* Virulence: Implication for Skin Homeostasis

**DOI:** 10.3389/fmicb.2016.00506

**Published:** 2016-04-15

**Authors:** Awa N'Diaye, Lily Mijouin, Mélanie Hillion, Suraya Diaz, Yoan Konto-Ghiorghi, Giuseppe Percoco, Sylvie Chevalier, Luc Lefeuvre, Nicholas J. Harmer, Olivier Lesouhaitier, Marc G. J. Feuilloley

**Affiliations:** ^1^Laboratory of Microbiology Signals and Microenvironnement LMSM, EA 4312, Normandie Université, Université de RouenEvreux, France; ^2^Department of Biosciences, University of ExeterExeter, UK; ^3^GlycoMev EA 4358, Normandie Université, Université de RouenMont-Saint-Aignan, France; ^4^Bio-EC LaboratoryLongjumeau, France; ^5^Dermatologic Laboratories UriageNeuilly-Sur-Seine, France

**Keywords:** skin bacterial communication, substance P, cathelicidin, human β-defensin 2, secretome, biofilm, thermo unstable ribosomal elongation factor

## Abstract

*Staphylococcus aureus* and *Staphylococcus epidermidis* are two major skin associated bacteria, and Substance P (SP) is a major skin neuropeptide. Since bacteria are known to sense and response to many human hormones, we investigated the effects of SP on Staphylococci virulence in reconstructed human epidermis model and HaCaT keratinocytes. We show that SP is stimulating the virulence of *S. aureus* and *S. epidermidis* in a reconstructed human epidermis model. qRT-PCR array analysis of 64 genes expressed by keratinocytes in the response to bacterial infection revealed a potential link between the action of SP on *Staphylococci* and skin physiopathology. qRT-PCR and direct assay of cathelicidin and human β-defensin 2 secretion also provided that demonstration that the action of SP on bacteria is independent of antimicrobial peptide expression by keratinocytes. Considering an effect of SP on *S. aureus* and *S. epidermidis*, we observed that SP increases the adhesion potential of both bacteria on keratinocytes. However, SP modulates the virulence of *S. aureus* and *S. epidermidis* through different mechanisms. The response of *S. aureus* is associated with an increase in Staphylococcal Enterotoxin C2 (SEC2) production and a reduction of exolipase processing whereas in *S. epidermidis* the effect of SP appears mediated by a rise in biofilm formation activity. The Thermo unstable ribosomal Elongation factor Ef-Tu was identified as the SP-interacting protein in *S. aureus* and *S. epidermidis*. SP appears as an inter-kingdom communication factor involved in the regulation of bacterial virulence and essential for skin microflora homeostasis.

## Introduction

Skin is a complex ecosystem including yeasts, fungi, bacteria, and viruses (Kong and Segre, 2012) and it is estimated that one billion bacteria colonize each square centimeter of skin (Grice et al., 2008). Moreover, almost 25% of the microbial population is located deeply into the skin through hair follicles, sweat, and sebaceous glands (Lange-Asschenfeldt et al., 2011) and is in close contact with eukaryotic cells. It has been known since the end of the twentieth century that bacteria can sense a large range of eukaryotic communication and defense molecules (Lesouhaitier et al., 2009). These factors have multiple effects on bacteria and can regulate their growth, adhesion, invasion, virulence, and/or biofilm formation activity (Lesouhaitier et al., 2009). As skin is the largest neuroendocrine organ of the human body (Roosterman et al., 2006) and many cutaneous hormones, neurohormones, and defense peptides diffuse in sweat (Cizza et al., 2008) and upper epidermal layers (Severini et al., 2002), the skin bacterial microflora is exposed in permanence to these eukaryotic factors.

The principal skin neuropeptide, Substance P (SP), released by sensory skin primary afferent C-fibers (Severini et al., 2002) shows important variations of local concentration under the effect of pain, stress, and infection (Harrison and Geppetti, 2001; Nakano, 2004; O'Connor et al., 2004). This peptide of the tachykinin family is involved in the pathogenesis of numerous skin diseases of multifactorial origins, like atopic dermatitis (Hosokawa et al., 2009; Misery, 2011). It is currently suspected that some of the effects of SP are mediated through interaction with skin microflora (Feuilloley et al., 2015). Indeed, we demonstrated recently that SP can stimulate the biofilm formation activity of a cutaneous strain of *Pseudomonas fluorescens* (Hillion et al., 2014) and the cytotoxicity of *Bacillus cereus* (Mijouin et al., 2013). An effect of SP on two of the major skin associated bacteria, namely *Staphylococcus aureus* and *Staphylococcus epidermidis* was also suspected (Mijouin et al., 2013). To date, however, as the effect of SP on bacteria has only been investigated using cellular models, the pathophysiological meaning of these observations remained hypothetical. In addition, the potential mechanism of action of SP requires clarification. Indeed, the thermo unstable ribosomal elongation factor (Ef-Tu), a well-known “moonlighting” protein (Amblee and Jeffery, 2015), was found as a sensor for SP in *B. cereus* (Mijouin et al., 2013). However, it has been also hypothesized that SP could promote the release of antimicrobial peptides such as the cathelicidin LL37 and human β-Defensin 2 (HBD2) (Brogden, 2005; Hansen et al., 2006). Furthermore, SP could also act indirectly on the bacterial microflora through modulation of antimicrobial peptides secretion. Although the basal concentration of antimicrobial peptides in skin is low and under the minimal inhibitory bacterial concentration (Lai and Gallo, 2009), it has been observed that antimicrobial peptides are detected by bacteria at sub-lethal doses (Hancock and Scott, 2000) where they can induce an unexpected increase of virulence (Madi et al., 2013).

In the present study, the effect of SP on the virulence of *S. aureus* and *S. epidermidis* was investigated using a reconstructed human epidermis model and by qRT-PCR array targeting key proteins involved in the response to bacterial interaction with the skin. As the expression of some antimicrobial peptides was not induced, this study was completed by assay of LL37 and HDB2 secretion by keratinocytes and measurement of the effect of these antimicrobial peptides on the cytotoxic and biofilm formation activity of both *Staphylococci*. Furthermore, the effect of SP on *S. aureus* and *S. epidermidis* adhesion on keratinocytes, secretome, and biofilm production were determined. These data lead, to the conclusion that SP exerts a direct control on the virulence of *Staphylococci* of physiopathological relevance. The SP sensory protein in these bacteria was identified.

## Materials and methods

### Bacterial strains and culture conditions

*S. aureus* MFP03 and *S. epidermidis* MFP04 were isolated from the skin of healthy volunteers and were characterized by phenotypic, metabolic, MALDI-Biotyper whole proteome, and 16S ribosomal RNA gene sequencing techniques (Hillion et al., 2013). For confocal microscopic studies, bacteria were transformed using pTeTON-GFP plasmid (Sastalla et al., 2009), encoding for the green fluorescent protein (GFP) and for ampicillin resistance. Staphylococci were routinely grown at 37°C, in Luria-Bertani (LB) broth. For pre-treatment, bacteria were diluted in fresh LB and the peptides were added at the beginning of the log growth phase. Bacteria were collected at mid-log growth phase (i.e., after a mean of 5 h incubation). Preliminary studies were performed to control for the absence of effect of the peptides on their growth kinetics. Before application on reconstructed skin or keratinocytes, bacteria were harvested by centrifugation and washed with sterile physiological water to remove any trace of free peptide. The bacterial density and the absence of contamination were controlled by plating. Transformed strains were grown in the same conditions except that ampicillin (100 μg/mL) or kanamycin (100 μg/mL) were added. The viability of the bacteria in the eukaryotic medium and under the different culture conditions was determined in preliminary studies as a control. Substance P (SP), neurokinin A and the reversed sequence peptide of SP (SPrev) were obtained from Polypeptides (Strasbourg, France). LL37 and HBD2 were purchased from Innovagen (Lund, Sweden).

### Virulence assays on reconstructed human epidermis

The effect of SP and neurokinin A on the virulence of S. *aureus* and *S. epidermidis* was studied using a reconstructed human epidermis model (RHE, SkinEthic™). The SP N-C reversed sequence peptide (SPrev) was without effect on the virulence of both *Staphylococci* (*data not shown*) and was used as a control for the whole study. According to provider instructions, immediately after reception the culture medium of RHE was changed using SkinEthic™ growth medium and the tissues were allowed to stabilize for 22 ± 1 h at 37°C in 5% CO_2_ atmosphere. The viability of RHE was measured using the MTT reduction assay performed according to ESAC-ECVAM and OECD Draft Revised Guideline TG431 (ESAC-ECVAM, 2009; OECD, 2014). Briefly, cellular NAD(P)H-dependent metabolic activity was evaluated through measurement of the conversion of MTT tetrazolium dye [3-(4,5-dimethylthiazol-2-yl)-2,5-diphenyltetrazolium bromide] into insoluble purple formazan crystals. For each test, a volume of 10 μL of bacterial culture (10^7^ or 10^8^ CFU/mL) was applied on each reconstructed epidermis. Sodium dodecyl sulfate [SDS; 5% (w/v) in water] and phosphate buffer saline (PBS; pH = 7.4, 0.1 M) were used as negative and positive controls, respectively. RHE were incubated with bacteria for 24 h and then were rinsed three times with PBS to remove non adherent germs. Tissues were then incubated in 300 μL of MTT solution (1 mg/mL) for 180 ± 5 min at 37°C in 5% CO_2_ atmosphere. Formazan was recovered from the tissues by dissolving crystals in 1.5 mL isopropanol at room temperature for 120 ± 5 min. Tissue viability was determined by measurement of the optical density (OD) at 540 nm. Results were expressed as percentages of the negative control values measured after exposure of RHE to sterile PBS. Negative controls were validated if the mean OD_540_ was ≥1.2 and the standard deviation was ≤18%. Positive controls consisting in SDS 5% exposure were validated if the mean viability was <40%, and the standard deviation value was ≤18%. Controls were performed to verify the absence of interference of bacteria with the MTT assay.

### Gene expression in reconstructed human epidermis

The expression of 64 genes encoding for antimicrobial peptides production, immune immunity proteins, chemokines, cytokines and their receptors, prostaglandin synthesis, epidermis differentiation, proteases and metalloproteinases, proteins involved in phagocytosis, invasion, internatization, cellular stress, apoptosis, growth factor production, and seboregulation was analyzed by qRT-PCR array using customized micro-chips (Bioalternatives, Gencay, France; Table 1). The expression level of these genes by RHE in the presence of control or SP treated *S. aureus* and *S. epidermidis* was compared to that of cellular housekeeping genes used as reference. RHE were exposed to the bacteria as previously described. After rinsing in PBS, RHE total RNAs were extracted using the Sigma-Aldrich TRI Reagent® Protocol. RNA later (Life Technologies cat. AM7020) was used to protect and stabilize RNA. The Dynabeads® mRNA Purification Kit (Life Technologies cat. 61006) was used to purify total RNA preparations and select mRNA. For each condition and qRT-PCR array 6 μg of purified mRNA was used. RNA integrity was checked using an Agilent 2100 Bioanalyzer (Agilent Technologies, Inc.). The yield of the extracted RHE total RNA was assayed from 1 μl RNA in a 2100 BioAnalyzer using the protocol of the RNA 6000 Nano Pico kit (Agilent Technologies, Inc.). The RIN values of the RHE total RNA from triplicates were between 7.9 and 9.3 out of 10.

**Table 1:** **List of the 64 genes encoding proteins investigated by qRT-PCR microarrays and mRNA expression calculated as percentage of control genes expression**.

			***S. aureus***	***S. epidermidis***
Housekeeping proteins (References)	GAPDH	Glyceraldehyde-3-phosphate dehydrogenase	Control	Control
	RPS28	Ribosomal protein S28	Control	Control
	RPL13A	Ribosomal protein L13a	Control	Control
Antimicrobial peptides and innate immunity proteins	CAMP	Cathelicidin antimicrobial peptide	152.1	41.4
	DEFB1	Defensin, beta 1	167.1	52.2
	DEFB103A	Defensin, beta 103A	ND	ND
	DEFB4A	Defensin, beta 4A	199.5	11.9
	PI3	Peptidase inhibitor 3, skin-derived	115.3	74.2
	RNASE7	Ribonuclease, RNase A family, 7	71.8	96.9
	S100A7	S100 calcium binding protein A7	154.7	31.2
	HAMP	Hepcidin antimicrobial peptide	317.2	65.8
	TLR1	Toll-like receptor 1	80.7	180.9
	TLR2	Toll-like receptor 2	100.1	174.2
	TLR3	Toll-like receptor 3	351.7	78.2
	TLR4	Toll-like receptor 4	ND	ND
	S100A8	S100 calcium binding protein A8	213.8	55.1
	S100A9	S100 calcium binding protein A9	90.5	107.2
Chemokines	IL8	Interleukin 8	239.7	61.8
	CCL5	Chemokine (C-C motif) ligand 5	223.1	101.9
	CCL27	Chemokine (C-C motif) ligand 27	129.4	69.9
	CCL20	Chemokine (C-C motif) ligand 20	167.9	8.9
	CXCL1	Chemokine (C-X-C motif) ligand 1	259.7	35.1
	CXCL2	Chemokine (C-X-C motif) ligand 2	ND	ND
	CXCL6	Chemokine (C-X-C motif) ligand 6	ND	ND
	IL17C	Interleukin 17C	ND	ND
	CXCL3	Chemokine (C-X-C motif) ligand 3	ND	ND
	CXCL5	Chemokine (C-X-C motif) ligand 5	ND	ND
	CXCL10	Chemokine (C-X-C motif) ligand 10	66.9	348.3
Cytokines and cytokines receptors	IL4R	Interleukin 4 receptor	88.4	123.9
	IL13RA2	Interleukin 13 receptor, alpha 2	174.2	ND
	IL23A	Interleukin 23, alpha subunit p19	ND	ND
	IL6	Interleukin 6	ND	ND
	TSLP	Thymic stromal lymphopoietin	107.4	109.7
	IL1A	Interleukin 1, alpha	1088.6	62.1
	IL1B	Interleukin 1, beta	147.1	38.4
	IL24	Interleukin 24	ND	ND
	TNF	Tumor necrosis factor	82.2	215.1
Prostaglandin synthesis enzyme	PTGS2	Prostaglandin-endoperoxide synthase 2	79.6	118
Markers of differenciation and proliferation	FLG	Filaggrin	156.5	61.1
	LOR	Loricrin	150.6	77.7
	CDSN	Corneodesmosin	94.2	87.5
	KRT5	Keratin 5, type II	32	393
	KRT10	Keratin 10	128.9	56
	KRT19	Keratin 19, type I	115.3	95.9
	MKI67	Marker of proliferation Ki-67	106.2	174.5
Proteases and Metalloproteinases	ADAM17	ADAM metallopeptidase domain 17	54.7	119.2
	KLK5	Kallikrein-related peptidase 5	39.9	243.5
	KLK7	Kallikrein-related peptidase 7	141.4	65.5
	MMP1	Matrix metallopeptidase 1	215.6	211.4
	MMP3	Matrix metallopeptidase 3	322.8	51
	MMP9	Matrix metallopeptidase 9	68.6	530.2
Proteins involved in phagocytosis, invasion, and internalization	RAC1	Ras-related C3 botulinum toxin substrate 1	162	71.9
	ILK	Integrin-linked kinase	148	58.5
	ITGA5	Integrin alpha 5	245.1	420.5
	ITGB1	Integrin beta 1	114.9	102.03
	DPP4	Dipeptidyl-peptidase 4	121.6	228.7
	STIP1	Stress-induced phosphoprotein 1	57.6	154.4
Markers of cellular stress and apoptosis	ATF3	Activating transcription factor 3	53.6	207.7
	HMOX1	Heme oxygenase (decycling) 1	54.3	124.1
	TP53	Tumor protein p53	37.9	196.8
	TP63	Tumor protein p63	295.3	193.6
Growth factors and receptors, proteins of seboregulation	IGF1R	Insulin-like growth factor 1 receptor	65.1	148.9
	SRD5A1	Steroid 5α reductase, α polypeptide 1	89.5	77
	CSF2	Colony stimulating factor 2	210.5	56.4

*ND, Non Detectable*.

### Antimicrobial peptides assays

The production of antimicrobial peptides, namely cathelicidin LL37 and Human β-Defensin 2 (HBD2), by keratinocytes in response to exposure to SP (10^−6^ M) or SPrev (used at the same concentration as a control) and bacteria pre-treated with SP or SPrev (10^−6^ M) was studied using the HaCaT keratinocytes cell line (CLS, Eppelheim, Germany). HaCaT cells were grown at 37°C in 5% CO_2_ atmosphere in Dulbecco's modified Eagle's medium (DMEM, Lonza) containing 25 mM glucose and supplemented with 10% inactivated fetal bovine serum, 2 mM L-glutamine (Lonza) and antibiotics (penicillin 100 IU/mL and streptomycin 100 μg/mL). Cells were used between passages 41 and 65. They were seeded in 24 well plates at a final density of 5^*^10^5^ cells per well, and grown for 48 h before use. A minimum of 24 h before interaction with bacteria, cells were starved of antibiotics and fresh serum-free medium was added. Antimicrobial peptides were assayed using human cathelicidin (cat. CSB-EL004476HU) and β-Defensin 2 (cat. CSB-E13201h) Elisa kits according to the manufacturer's protocol (Cusabio, Wuhan Hi-tech Medical Devices Park, China).

### Cytotoxicity, adhesion, and invasion assays

The cytotoxic potential of the bacteria was determined by measurement of lactate dehydrogenase (LDH) release by HaCaT cells due to cytoplasmic membrane destabilization. HaCaT cells were infected with mid-log growth phase bacterial suspension at a bacterium-to-cell ratio of 10:1. As previously indicated, bacteria were carefully rinsed before use to remove any trace of free peptide. The amount of LDH released by HaCaT cells was determined after 15 h of incubation using the Cytotox 96 enzymatic assay (Promega, France; Picot et al., 2003). Control studies performed using bacteria alone showed that none of the strains used in the present study produced metabolites interfering with the assays. For adhesion assays, after incubation with bacteria cells were gently washed with DMEM and then disrupted with 0.9% (v/v) Triton X100 in physiological water. Viable bacteria (intra- and extra-cellular) were counted by plating on Tryptic Soy Agar (TSA). Invasion was quantified by the gentamicin protection assay as previously described (Mezghani-Abdelmoula et al., 2004).

### Secretome analysis

Supernatants of *S. aureus* and *S. epidermidis* exposed to SP or SPrev were obtained by centrifugation and filtration. Proteins were precipitated by addition of trichloroacetic acid on ice. These proteins were harvested by centrifugation, washed in cold acetone, dried at room temperature, and resuspended in rehydratation buffer as described (Barbey et al., 2012). The protein concentration was determined by Bradford assay. Proteins were separated on 12% w/v polyacrylamide gel. For 2D-gel electrophoresis, protein samples were first separated by isoelectric focusing (IEF) using pH 4 to 7 non-linear IEF strips (GE Healthcare). The strips were then transferred horizontally onto 12% polyacrylamide gels, covered with 0.5% agarose and the second dimension separation was run. Proteins were visualized by colloidal Coomassie Brilliant Blue G250 staining. Gel images were captured using a GS-800 densitometer (Bio-Rad Laboratories). Variations of spot intensity and distribution were studied using the Bio-rad PDQuest 2D® analysis software. Electrophoretic bands and spots of interest were dissected and submitted to in-gel trypsin digestion (Barbey et al., 2012) and analyzed by Matrix Assisted Laser Desorption Ionization Time-of-Flight mass spectrometry (MALDI-TOF/TOF) using an AutoFlex III mass spectrometer (Bruker Daltonics). The spectrometer was used in a positive/reflector mode. Samples were spotted to MTP 384 ground steel targets (Bruker Daltonics) using freshly prepared matrix solution composed of 2,5-dihydroxybenzoic acid (20 mg/mL) in a solution of trifluoroacetic acid and acetonitrile [0.1 and 50% (v/v) in water]. Each spectrum was established over an average of 500–1000 laser shots. The FlexAnalysis software generated an MS peak list which was submitted for peptide mass fingerprinting using the integrated software Biotools (Version 3.2). The NCBI data base was searched using the online MASCOT software and statistical sequences analyses were performed using the probability-based Mowse score.

### Biofilm formation studies

The effect the antimicrobial peptides and SP on the biofilm formation activity of *S. aureus* MFP03 and *S. epidermidis* MFP04 was investigated by the crystal violet technique and by confocal laser scanning microscopy using GFP-transformed strains. The crystal violet technique was adapted from O'Toole et al. (2000). An aliquot of bacterial culture adjusted to OD_580_ = 0.4 was layered in a polystyrene microtitration plate in presence or not of peptides. After 24 h of incubation at 37°C the bacterial suspension was removed and the wells were rinsed and the remaining bacteria were stained with crystal violet (0.1%) for 30 min. After rinsing again with distilled water, the dye was recovered and the OD_595_ was measured. For microscopic studies, bacteria were inoculated at OD_580_ = 0.08 into fresh LB medium and grown in the presence of the peptides. Bacteria were then washed and resuspended in sterile physiological water. The suspension was adjusted to an OD_580_ = 1 and poured in Petri dishes containing sterile glass slides. Glass slides were removed after 2 or 24 h incubation, washed, heat fixed, and immediately observed using a LSM 710 inverted confocal laser-scanning microscope (Zeiss, Germany). Three-dimensional (3D) images and orthocuts were obtained using Zen® 2009 software. Biofilm thickness was quantified with the same software.

### Identification of the substance P binding protein

Mid-log growth phase SP treated bacterial cultures were centrifuged at 7000 × *g* for 10 min at room temperature. The pellets were resuspended in 5 mL of lysostaphine (10 μg/mL). This mix was incubated for 1 h at 37°C with shaking at 180 rpm. Then, bacteria were centrifuged for 20 min at 7000 × g at 4°C. Each pellet was solubilised in 6 mL of non-denaturing lysis buffer (Tris-HCl 50 mM pH 8, EDTA 4 mM, NaCl 137 mM, glycerol 10% (v/v), Triton X-100 1% (v/v), Phenylmethylsulfonyl-fluoride 1 mM) and supplemented with a protease inhibitors cocktail (Boehringer, Reims, France). Four cycles of freezing/thawing were applied to the suspension (−80°C for 20 min/37°C for 10 min). Bacterial lysis was completed by sonication using short pulses (1 min) on ice. The cell lysate was centrifuged at 13,000 × g at 4°C for 10 min to remove unbroken cells and the supernatant was ultra-centrifuged at 35,000 rpm for 50 min to isolate the membrane fraction. Membrane proteins were then solubilized in Tris-HCl 50 mM pH 8, MgCl_2_ 10 mM, Triton X-100 2%, Phenylmethylsulfonylfluoride 1 mM supplemented with a protease inhibitors cocktail.

The SP binding sites was identified by immunoprecipitation using a technique adapted from Mijouin et al. (2013). Sixty microliters of G protein-coupled agarose beads (Millipore) and 1.5 μg SP monoclonal antibodies (Abcam, Paris, France) were incubated overnight at 4°C. Meanwhile, 1.5 mg of bacterial membrane protein extract was incubated with SP or SPrev (10^−6^ M) for 1 h at room temperature under slow orbital agitation. To reduce the non-specific binding, 50 μL of rabbit polyclonal serum was added. Unlabelled G protein-coupled agarose beads were added to remove non-specific complexes. The samples were incubated for 30 min at 4°C under slow orbital agitation. Unlabelled beads were removed by centrifugation (10,000 × g, 4°C, 10 min). The supernatant containing the SP bound ligand was then mixed to the SP antibody-associated beads and incubated for 1 h at room temperature under slow orbital agitation. Beads were collected, washed two times with 250 μL of non-denaturing lysis buffer. Then, 50 μL of Laemmli 2x buffer [Tris-HCl 200 mM pH 6.8, glycerol 45% (v/v), SDS 6% (v/v), β-mercaptoethanol 6% (v/v), and bromophenol blue 0.03% (w/v)] was added and boiled for 5 min to separate the ligand from the beads. To visualize SP-bound proteins, beads were removed by centrifugation (14,000 × g, 4°C, 5 min) and the supernatant was run on a 12% w/v polyacrylamide gel SDS-PAGE. Proteins were visualized by colloidal Coomassie Blue G250 staining (Sigma).

As Ef-Tu was previously identified as the SP-binding site in *B. cereus* (Mijouin et al., 2013), we studied the identity of the SP binding protein in *S. aureus* and *S. epidermidis* by Western-Blot using polyclonal anti-Ef-Tu antibodies. These antibodies were produced in rabbit by Biogalenys SAS (Miserey, France) by immunization against the full 397 amino acids sequence of *Pseudomonas aeruginosa* Ef-Tu (UniProtKB/Swiss-Prot: P09591). Ef-Tu was obtained recombinantly from *Escherichia coli* using a synthetic codon optimized gene for *P. aeruginosa* Ef-Tu (MWG Operon) containing the leader sequence MHHHHHHSSGVDLGTENLYFQ*S to provide a cleavable 6*His tag. This was cloned into the NcoI and HindIII restriction sites of the pET-Duet1 vector (Novagen) and transformed into *E. coli* Rosetta2 (DE3) (Novagen). Proteins from mid-log growth phase bacteria were purified at 4°C using a 1 mL HisTrap crude FF column and a Superdex 200 16/600 h column (GE Healthcare) associated to an ÄKTAxpress system (GE Healthcare). For Western-Blot, immunoprecipitation SDS-PAGE gels were transferred onto nitrocellulose membranes at 50 mA for 1 h in TRIS base (192 mM) transfer buffer containing glycine (14.5% g/L w/v) and methanol (20% v/v) using a BioRad Mini TransBlot Electrophoretic Transfer Cell system. Membranes were then air-dried and immersed for 2 h in blocking buffer [Tris buffer saline (TBS): 50 mM, 150 mM NaCl, 5% whole milk]. Membranes were incubated with the primary antibody raised against Ef-Tu (1:100 in blocking buffer) at room temperature for 2 h while shaking. After incubation, the blot was washed three times in 1x TBS supplemented with 0.05% (w/v) Tween 20 for 30 min and incubated for 1.5 h while shaking with secondary antibody diluted 1/5000 (goat anti-rabbit IgG alkaline phosphatase conjugate, Biorad). Electrophorectic bands were detected using an alkaline phosphotase conjugate substrate kit (Biorad). *P. aeruginosa* Ef-Tu was used as positive control, and QSDA, a N-acyl homoserine lactonase, was used as a negative control.

### Statistical analysis

All experiments were conducted independently at least three times at different days. The results are expressed as mean ± SEM and statistical differences were determined using the Student's *t*-test. Significant differences were noted as ⋆, ⋆⋆, and ⋆⋆⋆ for *p*-values < 0.05, < 0.01, and < 0.001, respectively. For each biofilm, the thickness was calculated from a minimum of 20 measures in different fields using the Zen® 2009 software (Zeiss). The mean biofilm thickness was quantified over three different experiments and the Student *t*-test was used to compare the means. SDS-PAGE electrophoresis, bidimensional electrophoresis, immunoprecipitation, and Western Blot figures are representative of three independent experiments.

## Results

### Substance P increases the virulence of *S. aureus* and *S. epidermidis* on reconstructed human epidermis

**Figure 1:**
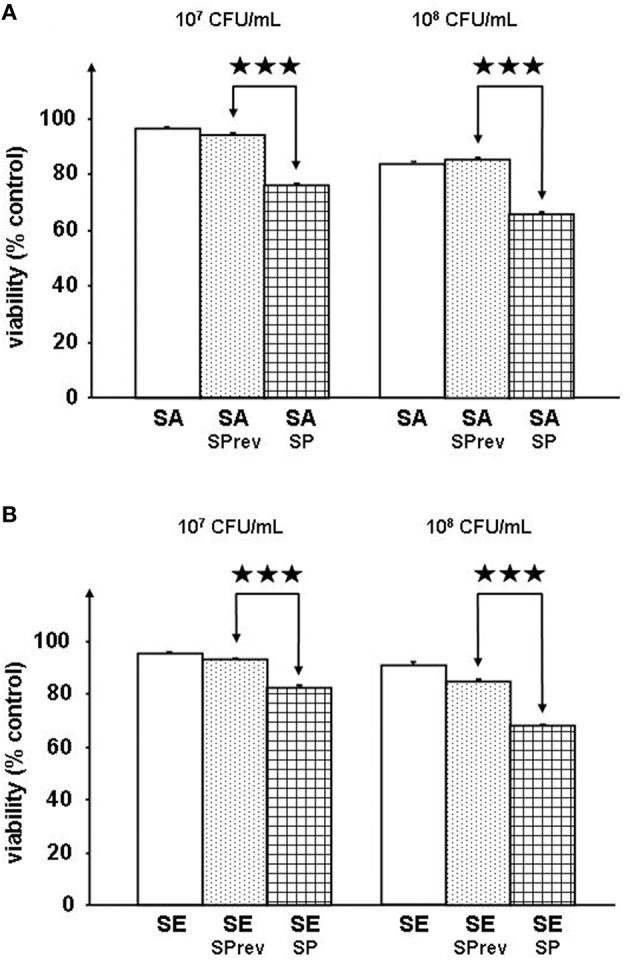
**Viability of reconstructed human epidermidis (RHE) exposed to *S. aureus* MFP03 (A) or *S. epidermidis* MFP04 (B) (10^7^ or 10^8^ CFU/mL) pre-treated with Substance P reverse (SPrev) used as a control or Substance P (SP) (10^−6^ M) (^⋆⋆⋆^*p* < 0.001)**.

Preliminary controls showed that exposure of *S. aureus* MFP03 and *S. epidermidis* MFP04 to SP (10^−6^ M) did not modify their growth kinetics (*data not shown*). The SP reversed sequence peptide (SPrev) was without effect on the virulence of *S. aureus* (Figure 1A) and *S. epidermidis* (Figure 1B) on RHE. This peptide (with the same amino acids composition as SP) was used as a control for the rest of the study allowing excluding any metabolic effect. When RHE were incubated in the presence of *S. aureus* (10^7^ or 10^8^ CFU/mL) pre-treated with SP (10^−6^ M) the MTT assay for cell metabolic activity revealed a significant decrease in the viability of the RHE (−19.36 ± 0.01% and −23.23 ± 0.01%, *p* < 0.001, respectively; Figure 1A). The effect of SP on *S. epidermidis* was similar with a reduction of RHE viability of 11.63 ± 0.01 and 19.70 ± 0.01% after incubation with bacterial culture (10^7^ or 10^8^ CFU/mL, *p* < 0.001, respectively; Figure 1B). Neurokinin A, another peptide of the tachykinin family also expressed in significant amount in skin (Roosterman et al., 2006), was totally without effect on *S. aureus* and *S. epidermidis* virulence *(data not shown)* when used at the same concentration as SP (10^−6^ M).

### Substance P treated bacteria affect the expression of proteins involved in host defense and inflammation in reconstructed human epidermis

**Figure 2:**
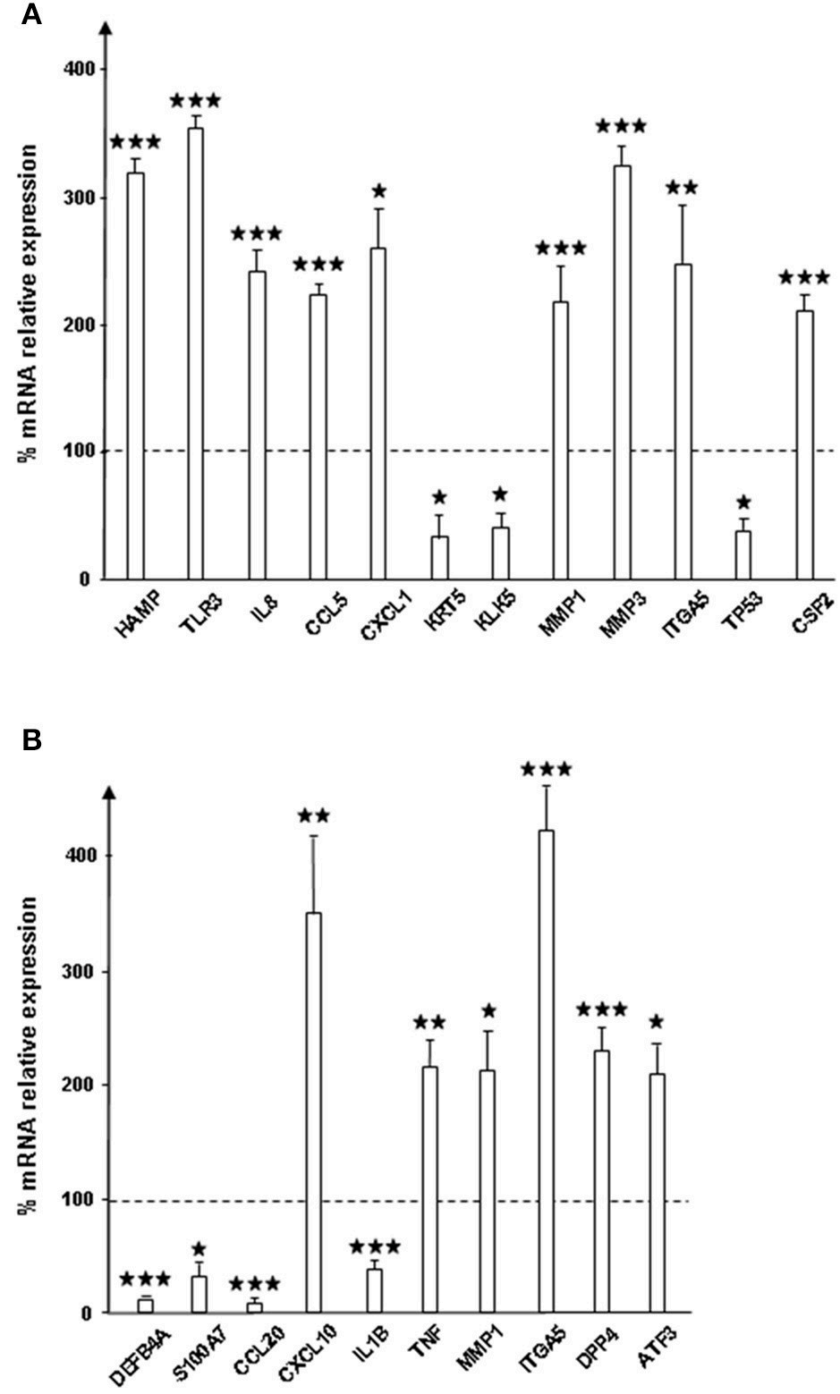
**Relative expression of genes encoding for response to infection and inflammation proteins by reconstructed human epidermidis (RHE) exposed to *S. aureus* MFP03 (A) or *S*. *epidermidis* MFP04 (B) pre-treated with Substance P**. Results are expressed as percentages to control RHE exposed to bacteria pre-treated with Substance P reverse (dotted line = 100%). Each value represents the mean ± SEM of three independent experiments. Only genes showing significant variation of expression on the 64 (^⋆^*p* < 0.05; ^⋆⋆^*p* < 0.01; ^⋆⋆⋆^*p* < 0.001). Names of proteins are given in Table 1.

RHE were exposed to control (SPrev) or SP treated bacteria, and expression changes of 64 genes was determined by qRT-PCR microarray (Table 1). Genes showing significant variations (>+200% for up-regulation and < −50% for down-regulation) are shown in Figure 2. SP-treated *S. aureus* led to alter expression of 12 RHE mRNAs, among which nine were over-produced [TLR3, one antimicrobial peptide (the hepcidin antimicrobial peptide), three chemokines (Interleukin 8, Chemokine ligands 1 and 5), two matrix metalloproteases (1 and 3), one cell contact protein (Integrin α5), and one growth factor (Colony stimulating factor 2), while three were under-produced (type II keratin 5, kallikrein-related peptide 1, and tumor protein 53)]. The maximal differences were obtained for TLR3 mRNA expression that reached +251 ± 17%, *p* < 0.001 (Figure 2A). In the case of *S. epidermidis*, expression of 10 genes was affected in RHE exposed to SP-treated bacteria (Figure 2B). Noticeably, only the two mRNAs encoding metalloprotease 1 (+111 ± 35%, *p* < 0.05) and integrin α5 (+320 ± 39%, *p* < 0.001), were increased similarly as in S. *aureus*. Four other mRNAs were also significantly over expressed, namely those encoding for chemokine ligand 10 (+248 ± 67%, *p* < 0.01), tumor necrosis factor (+115 ± 23%, *p* < 0.01), dipeptidyl-peptidase 4 (+129 ± 23%, *p* < 0.001), and activating transcription factor 3 (+108 ± 29%, *p* < 0.05). Conversely, the expression of defensin β4, S100 calcium binding protein A7, chemokine ligand 20, and interleukin 1β was reduced.

### Substance P stimulates the secretion of cathelicidin by keratinocytes but control and SP treated bacteria have limited effects on antimicrobial peptides

**Figure 3:**
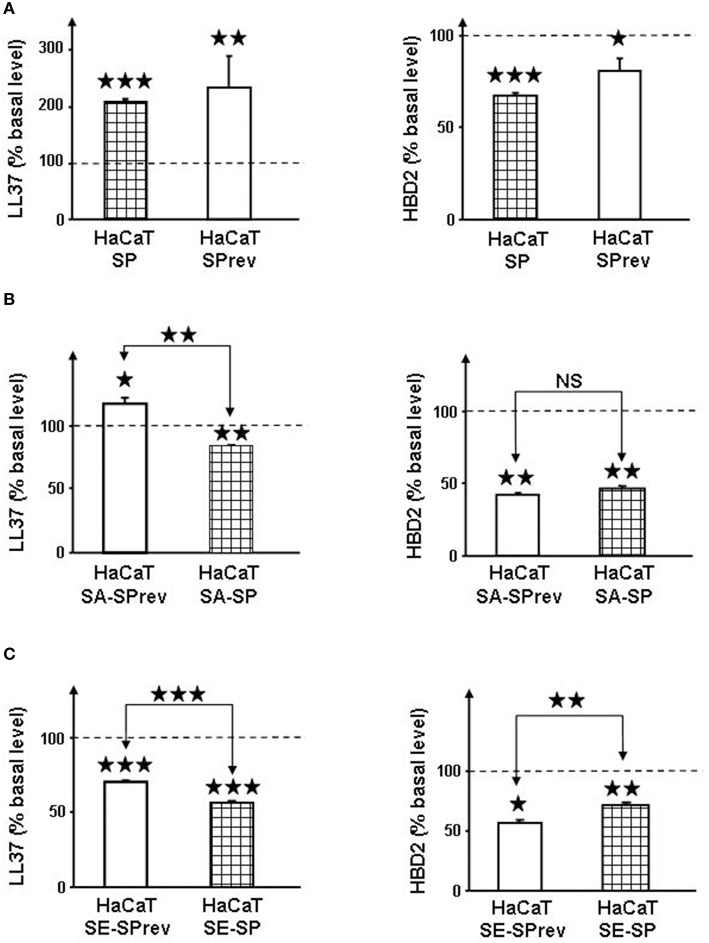
**Effect of Substance P (SP) and Substance P reverse (SPrev) (A), Substance P reverse (SA-SPrev) and Substance P treated *S. aureus* (SA-SP) (B) and Substance P reverse (SE-SPrev) and Substance P treated *S. epidermidis* (SE-SP) (C) on cathelicidin LL37 and β-defensin 2 production by HaCaT keratinocytes**. On each figures, dotted lines indicate the basal production of LL37 and HBD2 by HaCaT in the absence of treatment (control). Each value is the mean ± SEM of three independent experiments. (NS = non significant; ^⋆^*p* < 0.05; ^⋆⋆^*p* < 0.01; ^⋆⋆⋆^*p* < 0.001).

The effect of SP and of SP-treated *S. aureus* and *S. epidermidis on* cathelicidin LL37 and β-defensin 2 (HBD2) productions by HaCaT keratinocytes was studied. By itself SP (10^−6^ M) alone induced a marked increase in LL37 secretion (+212 ± 2%, *p* < 0.001; Figure 3A). Conversely a decrease in HBD2 was observed (−32 ± 1%, *p* < 0.01). Unexpectedly, exposure of HaCaT cells to SPrev alone lead to similar results with a rise of LL37 (+237 ± 20%, *p* < 0.01) and a reduction of HBD2 production (−19 ± 6%, *p* < 0.05). When keratinocytes were exposed to *S. aureus* pre-treated with SP (10^−6^ M) a decrease in LL37 and HBD2 secretion (−15.8 ± 0.7%, *p* < 0.05 and −54 ± 2%, *p* < 0.01, respectively) was noted (Figure 3B). Similar results were observed when HaCaT cells were exposed to SP pre-treated *S. epidermidis*, with a reduction of antimicrobial peptides secretion that reached −42.8 ± 0.8% (*p* < 0.001) for LL37 and −29 ± 2% (*p* < 0.01) for HBD2 (Figure 3C). However, when HaCaT cells were exposed to control SPrev-treated *S. aureus*, there was an increase in LL37 secretion. In all other controls, there was a reduction in antimicrobial peptides secretion, as was observed with SP-treated micro-organisms.

### Cathelicidin and β-defensin 2 have no effect on the cytotoxicity on *S. aureus* and decrease that of *S. epidermidis*

**Figure 4:**
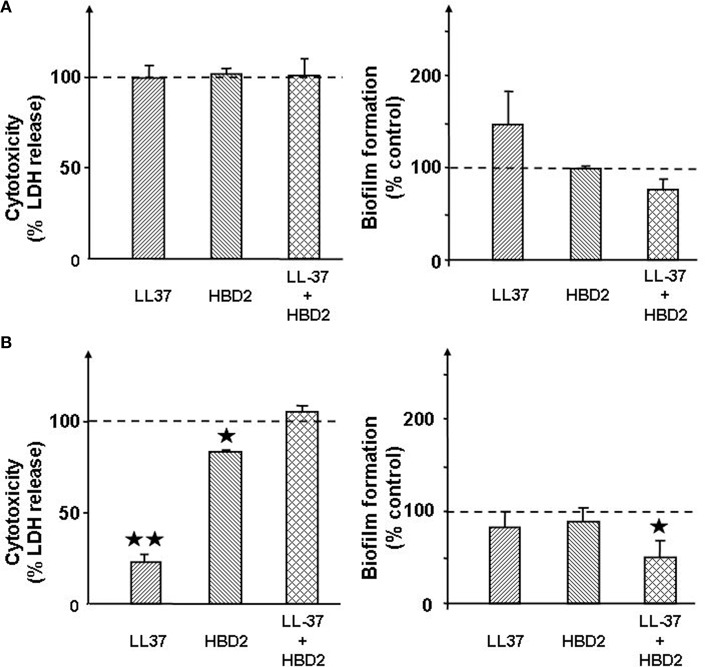
**Effect of the cathelicidin LL37 (1 μg/mL) and of β-defensin 2 (HBD2) (1 μg/mL) alone or in association on the cytotoxicity and biofilm formation activities of *S. aureus* MFP03 (A) and *S. epidermidis* MFP04 (B)**. Dotted lines indicate the basal cytotoxicity and biofilm formation activity of bacteria in the absence of treatment (control). Each value is the mean ± SEM of three independent experiments. (^⋆^*p* < 0.05; ^⋆⋆^*p* < 0.01).

Preliminary studies showed that LL37 and HBD2 at a concentration of 1 μg/mL alone or in association were without effect on the growth of *S. aureus* and *S. epidermidis (data not shown)*. Both antimicrobial peptides were totally without effect of the cytotoxic activity of *S. aureus* on HaCaT cells (Figure 4A). In the absence or presence of LL37 and/or HBD2 only non-significant variations of biofilm formation were observed with *S. aureus*. Conversely, treatment of *S. epidermidis* with LL37 (1 μg/mL) resulted in a marked reduction of cytotoxicity (−77 ± 4%, *p* < 0.01; Figure 4B). Exposure of *S. epidermidis* to HBD2 showed a more marginal, but significant, decrease in bacteria cytotoxicity on HaCaT cells (−17 ± 1%, *p* < 0.05). However, when the two peptides were used in association, their individual effects on *S. epidermidis* cytotoxicity were totally abolished. Similarly to *S. aureus*, neither LL37 nor HBD2 treatments were leading to significant altered biofilm formation. However, association of both peptides led to slightly reduce biofilm production of *S. epidemidis*.

### Substance P increases adhesion of *S. aureus* and *S. epidermidis* on keratinocytes

**Figure 5:**
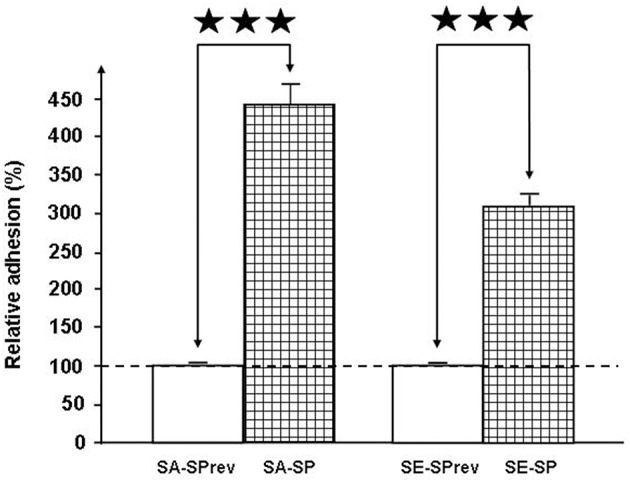
**Effect of Substance P (SP) and Substance P reverse (SPrev) (10^−6^ M) on the adhesion properties of *S. aureus* MFP03 and *S*. *epidermidis* MFP04 on HaCaT keratinocytes**. The dotted line indicates the basal adhesion of bacteria (100%) in the absence of treatment (control). Each value is the mean ± SEM of three independent experiments (^⋆⋆⋆^*p* < 0.001).

The gentamicin protection assay was used to obtain a measurement of total cell-associated bacteria. This revealed that SP strongly increased the adhesion potential of *S. aureus* and *S. epidermidis* on HaCat keratinocytes (+438 ± 24%, *p* < 0.001 and +307 ± 13%, *p* < 0.001, respectively; Figure 5). Conversely, the invasion potential of both *Staphylococci* remained unchanged (*data not shown*). Used at the same concentration (10^−6^M), SPrev had no effect on the adhesion potential of the two bacteria.

### Substance P promotes the release of enterotoxin C2 and blocks exolipase processing in *S. aureus*

**Figure 6:**
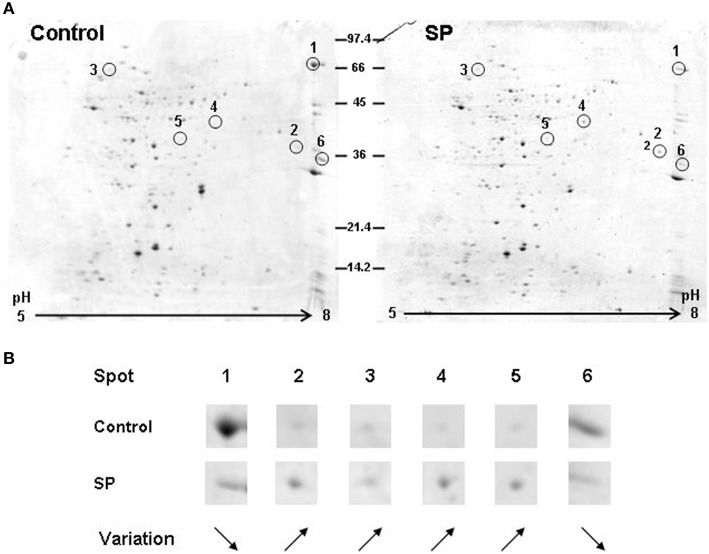
**Bidimensional electrophoresis analysis of secreted *S. aureus* MFP03 proteins after 1 h treatment with Substance P reverse (Control) or Substance P (SP) (10^−6^ M). (A)** Six spots were modified after exposure of the bacteria to SP. **(B)** Two proteins (1 and 6) were markedly decreased whereas the expression of four others was upregulated. Proteins corresponding to these spots are presented in Table 2. Results are representative of three independent experiments.

The secretome from mid-log growth phase cultures of *S. aureus* and *S. epidermidis* corresponding to the conditions of the adhesion assays were studied by 2D-gel electrophoresis. Image analysis of three replicates of 2-D gels allowed the detection of intensity variations in six major spots between control and SP treated *S. aureus* (Figure 6A). Two of these spots (1 and 6) were down regulated proteins, whereas the four others are over produced in response to SP (Figure 6B). Spots 1 and 4 were identified by MALDI-TOF/TOF analysis as the staphylococcal 77 kDa lipase and the 44 kDa lipase precursor, respectively. Spot 2 was identified as the 32 kDa staphylococcal enterotoxin C2. As presented in Table 2, the three other spots were proteins involved in folding and potential intermediate metabolism enzymes. The secretome of *S. epidermidis* showed a limited number of exoproteins and no difference was observed between control and SP-treated bacteria.

**Table 2:** **Proteins under- and over-expressed in the secretome of Substance P treated *S*. *aureus* MFP03 identified by MALDI-TOF/TOF**.

**Spot number**	**NCBl accession number**	**Gene**	**Putative function**	**Protein domain(s)**	**Mascot score**	**Nb of matched peptides**	**Coverage (%)**	**Mass(Da) & pI**
1	EEV69102		Lipase	Predicted acetyltransferase and hydrolase with α/β hydrolase fold domain	274	39	52	76557/8.38
2	EFK82013	sec4	Staphylococcal enterotoxin C2	Staphylococcal/Streptococcal toxin 0B-fold and grasp domain	156	18	56	32654/7.6
3	YP_0057 44993	tig	Prolyl isomerase Protein export (chaperone)		126	12	29	48893/5.35
4	A8W37176		Lipase precursor	Esterase lipase domain	135	14	37	44329/6.83
5	NP372219	citZ	Citrate synthase	Coenzyme A binding site, oxalacetate citrate binding site, catalytic triad	205	18	39	42566/6.41
6	YP_500307		Hypothetical protein SAOUHSC_01802	Oxalacetate citrate binding site, coenzyme A binding site, catalytic triad	116	13	33	3237117.17

### Substance P increases the biofilm formation activity of *S. epidermidis*

**Figure 7:**
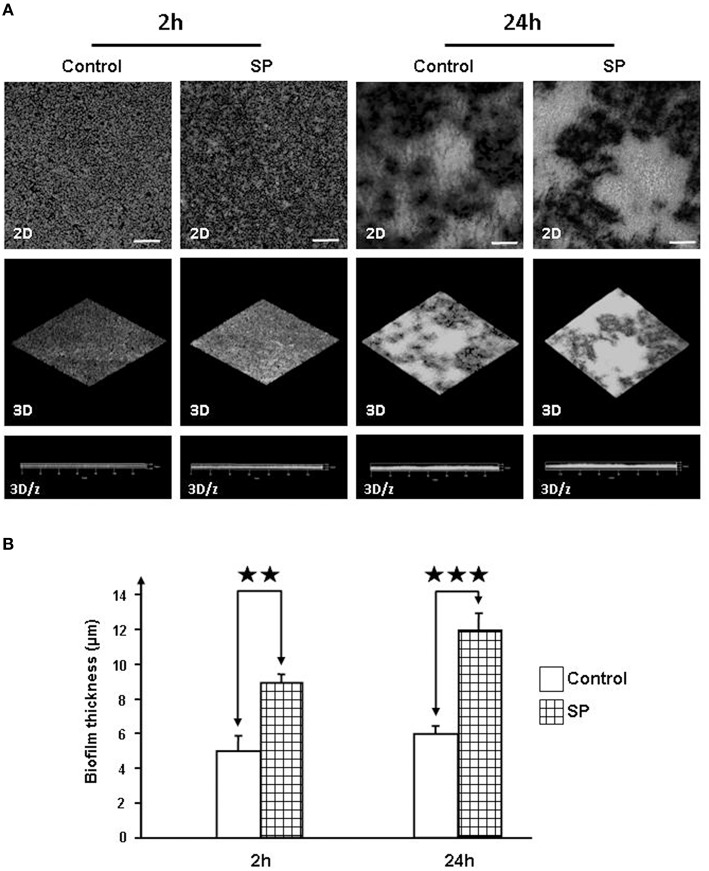
**Effect of Substance P reverse SPrev (Control) and Substance P (SP) (10^−6^ M) on the biofilm formation activity of *S. epidermidis* MFP04**. The biofilm formation activity of bacteria was observed after 2, 5, and 24 h. **(A)** Two dimensions (2D), reconstructed three-dimensions (3D) and ortho cuts (3D/z) images. Pictures are representative of three independent experiments. **(B)** Evolution of the thickness of the biofilm after 2 and 24 h incubation with SP. Each value represents the mean ± SEM of three independent experiments (^⋆⋆^*p* < 0.01; ^⋆⋆⋆^*p* < 0.001).

The biofilm formation activity of *Staphylococci* was investigated in static conditions on glass slides by confocal laser scanning microscopy. Biofilm production by *S. aureus* MFP03 was very limited and remained unchanged in the presence of SP. In contrast, after a 2 h treatment with SP (10^−6^ M), the mean surface coverage of SP-treated *S. epidermidis* MFP04 was markedly increased (Figure 7A). Both density and thickness of the biofilm were higher. As calculated using the Zen® software, after a 2 h exposition to SP the mean thickness of the biofilm evolved from 5 to 9 μm (Figure 7B). This effect was maintained after 24 h exposition to SP with a mean biofilm thickness of 6 μm for control and 12 μm for SP treated bacteria (Figure 7B).

### The ribosomal elongation factor Ef-Tu acts as a substance P binding protein in *Staphylococci*

**Figure 8:**
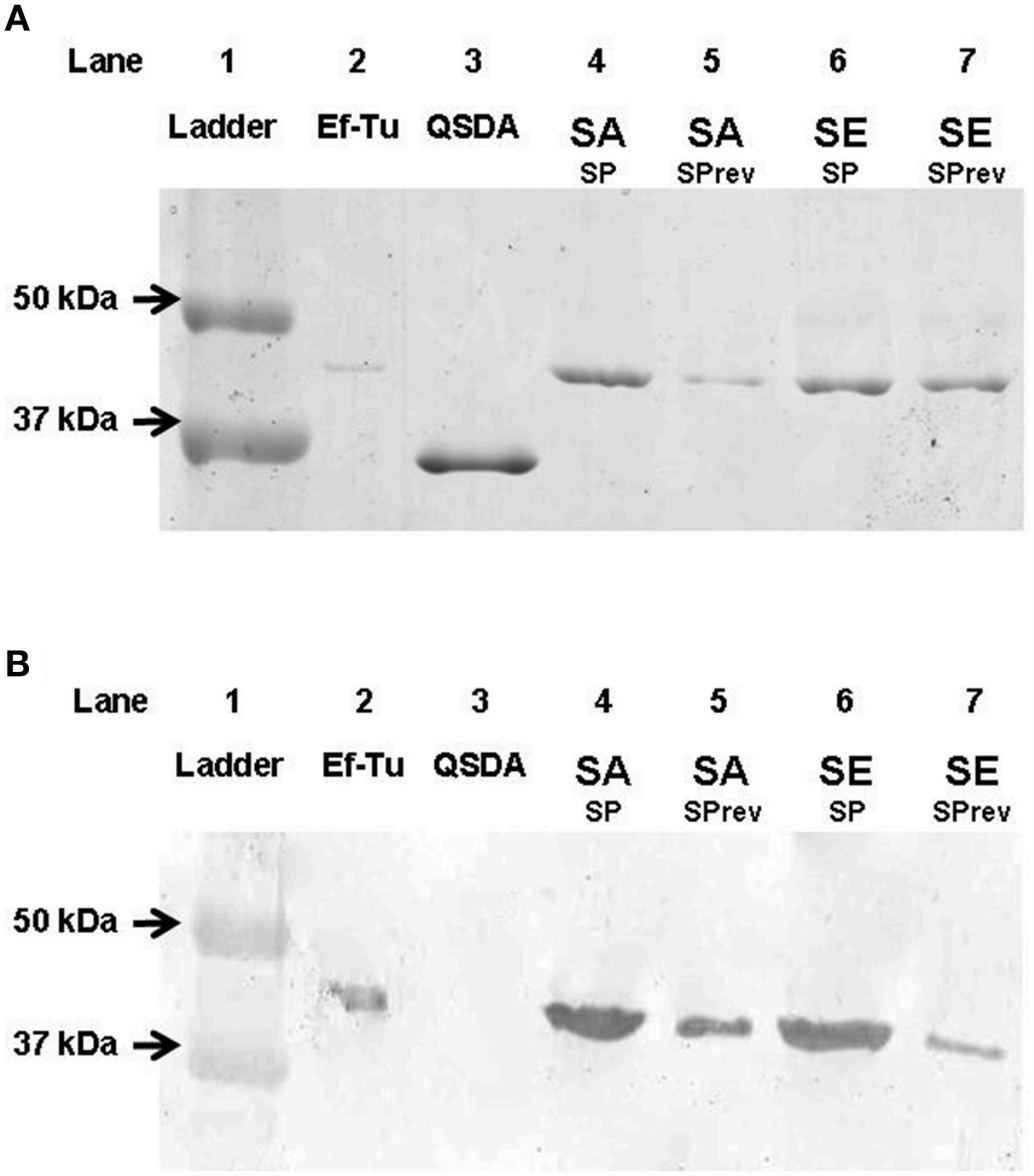
**SDS-PAGE analysis of *S. aureus* and *S. epidermidis* Substance P (SP) binding proteins separated by immunoprecipitation and labeled by colloidal Coomassie Blue G250 staining (A) and identified by western blot after transfer on nitrocellulose gel using *P. aeruginosa* Ef-Tu polyclonal antibidodies (B)**. Line 1, ladder; Line 2, *P. aeruginosa* Ef-Tu (mass marker and positive control for western blot); Line 3, QSDA protein (negative control for western blot); Line 4, Substance P treated *S. aureus* membrane extracts (SA SP); Line 5, Substance P reverse treated *S. aureus* membrane extracts (SA SPrev); Line 6, Substance P treated *S. epidermidis* membrane extracts (SE SP), Line 7: Substance P reverse treated *S. epidermidis* membrane extracts (SE SPrev). Results are representative of three independent experiments.

The presence of a SP binding site was investigated in the membrane of *S. aureus* and *S. epidermidis*. The properties of SP (ionic charge and structure) limit its potential penetration in bacteria. Furthermore, a membrane form of Ef-Tu has previously been identified as the SP receptor in *B. cereus* (Mijouin et al., 2013). A band, corresponding to a 43 kDa protein, was found by immunoprecipitation as a ligand of SP in *S. aureus* and *S. epidermidis* membrane extracts (Figure 8A). Binding of SPrev on the same 43 kDa protein, was also observed in *S. aureus* and *S. epidermidis* membrane extracts but with a lower intensity. Nevertheless, SPrev is an inverted peptide (i.e., the sequence is not scrambled), and some epitopes of native SP may be preserved. Identification of the 43 kDa SP binding molecule as the Ef-Tu protein was demonstrated by western blot using an antibody raised against *P. aeruginosa* Ef-Tu (73% sequence identity to Ef-Tu from *S. aureus* and *S. epidermidis*; **Figure 8B)**. The identity of Ef-Tu as the SP binding protein was confirmed by MALDI-TOF analysis of SDS-PAGE SDS bands with MASCOT scores >98 and a coverage percentages >60%.

## Discussion

In the present work, we show that SP, at a concentration in the same range as potentially present in sweat or in intradermal areas (Ostlere et al., 1995; Cizza et al., 2008) promotes the virulence of *Staphylococci* on reconstructed human epidermidis (RHE). RHE is a simplified skin model in which keratinocytes are grown on an artificial matrix but, as with human epidermidis, they show a fully differentiated stratum corneum (Pendaries et al., 2015). They are therefore, close to the behavior of human skin explants, without the drawback of their variability and the presence of remaining commensal germs that are almost impossible to eliminate. qRT-PCR arrays reveal that SP-treated *S. aureus* and *S. epidermidis* induced a significant and specific response of RHE. Treatment of *S. aureus* by SP was leading to up-regulation of the expression of at least nine mRNA, among which the one showing the main increase was encoding TLR3, a protein involved in pathogen-associated molecular patterns (PAMPs) recognition (Lebre et al., 2007). The associated over-expression of chemokines (CCL5 and CXCL1) is consistent with an increase in the virulence of SP treated *S. aureus* and the up-regulation of interleukin 8 (IL8) suggests that an inflammatory response in RHE has been activated, as is observed in skin (Nedoszytko et al., 2014). Hepcidin antimicrobial peptide (HAMP) modulates iron storage and its increase also suggests inflammation pathways are being activated (Andrews, 2004). HAMP has a broad antimicrobial spectrum but *S. aureus* is considered almost insensitive to this peptide (Lombardi et al., 2015) suggesting that the effect of SP on *S. aureus* is independent of HAMP. Nevertheless, it is interesting to note that SP-treated *S. aureus* did not induce the expression of any other studied antimicrobial peptide. The response of RHE to SP-treated *S. epidermidis* is notably different. Integrin α5, the principal protein whose expression was increased, is involved in *S. aureus* (Alva-Murillo et al., 2014) and *Streptococci* (McFadden et al., 2009) internalization leading to cutaneous lesions associated to psoriasis (McFadden et al., 2009). It has been also recently demonstrated that over-expression of the chemokine ligand 10 (CXCL10), as was observed here with of SP-treated *S. epidermidis*, is clearly associated with psoriasis (Ferrari et al., 2015). It is too soon to postulate a link between the effect of SP on *S. epidermidis* and *S. aureus* and psoriasis, but this hypothesis is worthy consideration.

It was postulated that SP could act indirectly on bacteria through activation of LL37 and HBD2 secretion (Brogden, 2005; Hansen et al., 2006). However, these antimicrobial peptides are either secreted by cleavage from a precursor or require induction delay making qRT-PCR poorly adapted to their study. Their expression was therefore followed by immunoassay. We observed that SP indeed stimulates LL37 production, but SPrev has a similar effect, suggesting that this response is poorly specific and that structural homologies between these peptides are sufficient to activate the keratinocytes response. Conversely SP and SPrev provoked a marginal but significant reduction of HBD2 secretion. A potential effect of SP-treated bacteria on LL37 and HDB2 secretion was then investigated. Although significant differences of response amplitude were noted, control and SP-treated bacteria had globally the same effects. Moreover, all *Staphylococci* exposed to SP showed a significant decrease of LL37 or HDB2 secretion. The study of a potential effect of SP on bacteria through antimicrobial peptides secretion was completed by determination of the effect of LL37 and HBD2 on the cytotoxicity and biofilm formation activity of *S. aureus* and *S. epidermidis*. Alone or in association, these antimicrobial peptides were without significant effect on *S. aureus* activity. In the case of *S. epidermidis* a decrease of cytotoxicity and biofilm formation was observed. Taken together, the present data demonstrate that the effect of SP on the virulence of *Staphyloccoci* is not mediated through activation of antimicrobial peptide secretion.

To get further insights into the role of SP in promoting *Staphyloccoci* virulence, we next investigated the effects of SP on the adhesion and invasion potential of bacteria. Exposure of both *Staphylococci* to SP resulted in a marked increase of their adhesion potential on keratinocytes whereas their invasive activities were unchanged. Adhesion is a key step in internalization and we cannot exclude that invasion occurs later or an effect of SP-treated *Staphylococci* on the internalization of other bacterial species such as was observed by exposure of cells to Eap, the multifunctional extracellular adherence protein of *S. aureus* (Bur et al., 2013). Further studies, by analysis of the secretome and biofilm formation activity of *S. aureus* and *S. epidermidis*, revealed that the two bacteria have very different reactions to SP, albeit they all act in the same sense of an increase of their infectious potential. Indeed, whereas SP had no effect on the release of exoproteins by *S. epidermidis*, SP stimulated the production of the Staphylococcal enterotoxin C2 by *S. aureus* MFP03. It is particularly interesting to note that Staphylococcal enterotoxins are members of a family of bacterial toxins that are increased in *S. aureus* strains associated with atopic dermatitis (Nada et al., 2012), psoriasis (Atefi et al., 2014), and even diabetic foot ulcers (Vu et al., 2014). In parallel, in *S. aureus* SP induced a decrease in the production of a lipase, whereas the lipase precursor accumulated suggesting that under the influence of SP, the precursor was not processed. We cannot exclude that this reaction had also an indirect effect on the virulence of *S. aureus*. Indeed, if the lipase activity is reduced, this can favor an accumulation of lipids on the skin surface. A hydrophobic environment is particularly important for the growth of this bacterium (Wright et al., 2004). In addition, *S. aureus* is sensitive to long chain unsaturated free fatty acids, which act as a barrier to its colonization and infection and exert a direct antimicrobial activity (Kenny et al., 2009). In the case of *S. epidermidis*, whose secretome was not modified by SP, the peptide stimulated the biofilm formation activity. In fact, the ability of *S. epidermidis* to produce biofilms on biotic surfaces, such as skin, constitutes its primary virulence factor and allows its dissemination in deep tissues and through bloodstream (Vuong and Otto, 2002).

It is not clear why Staphylococci have evolved or preserved an SP detection system. The lack of effect of the SP reversed sequence peptide and of a closely related tachykinin, Neurokinin A, shows the high level of specificity of the recognition system. In *B. cereus*, SP was shown to bind on Ef-Tu (Mijouin et al., 2013). Ef-Tu is a protein that is highly conserved throughout bacterial evolution and its name is excessively restrictive in regard of its multiple known functions. Indeed, Ef-Tu is produced in large excess in regard to its stoechiometric association to ribosomes and it was postulated that in stress conditions Ef-Tu translocates to the bacterial membrane where it changes of conformation and acquires new roles (Dallo et al., 2002, 2012). *Staphylococci* also express Ef-Tu and in *S. aureus* Ef-Tu is found not only in the cytoplasm but also in the cell envelope (Gatlin et al., 2006). In the present study we observed that, as in *B*. cereus (Mijouin et al., 2013), in *S. aureus* and *S. epidermidis*, SP was binding to Ef-Tu. This suggests that Ef-Tu acts as an SP sensor in many Gram-positive bacteria, and even potentially in other eubacteria. Indeed, *P. fluorescens*, a typical Gram-negative bacterium, is also capable of recognizing SP (Hillion et al., 2014). Although the SP binding site was not identified in this species, Ef-Tu is also suspected to act as a sensor in *Pseudomonas* (Dagorn et al., 2013). Previous studies have shown that in different bacterial species Ef-Tu is also acting as a membrane binding protein for fibrinogen (Kunert et al., 2007) or plasminogen (Chung et al., 2011). The present results extend the spectrum of Ef-Tu as “moonlighting protein” exerting different functions in the bacterial cytoplasm and in the membrane (Amblee and Jeffery, 2015).

The present study demonstrates that the effects of SP on the virulence, adhesion and biofilm formation activities of *S. aureus* and *S. epidermidis* are of potential pathophysiological relevance. We confirm the hypothesis that SP stimulates LL37 secretion. However, the action of SP on bacteria appears independent of antimicrobial peptides. SP acts on *S. aureus* and *S. epidermidis* through different mechanisms although it can be recognized at least by the same sensor protein, Ef-Tu. This confirms that SP acts as an inter-kingdom communication factor involved in the regulation of skin microflora homeostasis.

## Author contributions

Conceived and designed experiments: LM, AN, MH, SC, MF. Performed the experiments: LM, AN, MH, SD. Analyzed the data: LM, AN, MH, SD, YK, GP, SC, LL, OL, MF. Contributed reagents/material/analysis tools: SD, GP, NH. Wrote the paper: LM, AN, YK, SC, OL, MF. All authors read and approved the final manuscript.

### Conflict of interest statement

The authors declare that the research was conducted in the absence of any commercial or financial relationships that could be construed as a potential conflict of interest. GP is employed by Bio-EC Laboratory and LL by the Dermatologic Laboratories Uriage.
